# A Qualitative Study of Vaccine Acceptability and Decision Making among Pregnant Women in Morocco during the A (H1N1) pdm09 Pandemic

**DOI:** 10.1371/journal.pone.0096244

**Published:** 2014-10-14

**Authors:** Anna-Leena Lohiniva, Amal Barakat, Erica Dueger, Suzanne Restrepo, Rajae El Aouad

**Affiliations:** 1 Global Disease Detection Center–Egypt, US Naval Medical Research Unit no. 3, Cairo, Egypt; 2 Centre National de réfèrence Grippe–Institut National d'Hygiène–Ministry of Health, Rabat, Morocco; 3 Centers for Disease Control and Prevention, Atlanta, Georgia, United States of America; and Global Disease Detection Center–Egypt, US Naval Medical Research Unit no. 3, Cairo, Egypt; University of Chieti, Italy

## Abstract

Vaccination uptake of pregnant women in Morocco during the A (H1N1) pdm09 pandemic was lower than expected. A qualitative study using open-ended questions was developed to explore the main determinants of acceptance and non-acceptance of the monovalent A (H1N1) pdm09 vaccine among pregnant women in Morocco and to identify information sources that influenced their decision-making process. The study sample included 123 vaccinated and unvaccinated pregnant women who were in their second or third trimester between December 2009 and March 2010. They took part in 14 focus group discussions and eight in-depth interviews in the districts of Casablanca and Kenitra. Thematic qualitative analysis identified reasons for vaccine non-acceptance: (1) fear of the monovalent A (H1N1) pdm09 vaccine, (2) belief in an A (H1N1) pdm09 pandemic conspiracy, (3) belief in the inapplicability of the monovalent A (H1N1) pdm09 vaccine to Moroccans, (4) lack of knowledge of the monovalent A (H1N1) pdm09 vaccine, and (5) challenges of vaccination services/logistics. Reasons for vaccine acceptance included: (1) perceived benefits and (2) modeling. Decision-making was strongly influenced by family, community, mass media, religious leaders and health providers suggesting that broad communication efforts should also be used to advocate for vaccination. Meaningful communication for future vaccine campaigns must consider these context-specific findings. As cultural and religious values are shared across many Arab countries, these findings may also provide valuable insights for seasonal influenza vaccine planning in the Middle East and North Africa region at large.

## Introduction

During the A (H1N1) pdm09 pandemic, the World Health Organization (WHO) recommended that pregnant women receive the monovalent A (H1N1) pdm09 vaccine during pregnancy [1] in accordance with studies indicating increased morbidity and mortality among pregnant women associated with the A (H1N1) pdm09 infection [2]–[4]. Furthermore, influenza vaccines are generally safe for both mothers and their fetus [5], [6]. In fact, studies show that infants may receive protection from the virus through the transfer of antibodies from mothers vaccinated with influenza vaccine [7]. However, despite WHO recommendations, uptake of the monovalent A (H1N1) pdm09 vaccine among pregnant women worldwide remained lower than expected during the pandemic response (2009–2010) [8], [9], [10].

In Morocco (population 32 million), the monovalent A (H1N1) pdm09 vaccine was first made available to pregnant women in December 2009 at the Ministry of Health (MoH) facilities with 4,050,000 doses purchased for the target population of healthcare workers and other “high-risk groups” including pregnant women. Concurrently, a pandemic influenza awareness campaign was launched to inform the public about the monovalent A (H1N1) pdm09 vaccine with a key objective to highlight the benefits of vaccination for pregnant women. Despite these campaigns, only 167,870 (41%) of pregnant women were vaccinated.

Studies from other countries among various audiences including pregnant women have identified various barriers to the uptake of the monovalent A (H1N1) pdm09 vaccine including perceived risks and safety concerns [9], [11]–[23]. In addition, studies among pregnant women have identified pregnancy and fetus-specific concerns as barriers to the uptake of the vaccine [11], [12], [17]. Most of the previous studies are based on quantitative approaches, however, qualitative methodologies have provided an important opportunity to understand vaccine-associated perceptions and decision making [24]. There are no previous qualitative studies describing monovalent A (H1N1) pdm09 vaccine uptake among pregnant women in Morocco or in the Arab region. This study aimed to describe perceptions of pregnant women in Morocco related to the A (H1N1) pdm09 infection, to identify factors that encouraged or discouraged them from taking the monovalent A (H1N1) pdm09 vaccine during the pandemic response (2009–2010), and the sources of information that influenced their decision-making process.

## Methods

### Site selection

The MOH selected health facilities located in the regions of Casablanca (Bernoussi, Ain Chock, Hassania and Dar Bouazza) and Kenitra (Sidi Yahya, Ben Mansour and Beb Fes) to undertake the study. Casablanca is Morocco's largest city and part of the predominantly urban region known as Grand Casablanca, while Kenitra is predominantly rural.

### Study design and tools

A qualitative exploratory study designed with open-ended questions was used to gain an in-depth understanding of underlying factors that influenced the uptake of the monovalent A (H1N1) pdm09 vaccine. Focus group discussions (FGDs) were utilized to create an environment where study participants could openly discuss their ideas, beliefs and perceptions about the vaccine uptake in a group setting. In-depth interviews (IDIs) were used to further corroborate the comments made in the FGDs by obtaining narrative accounts of participants' experiences related to the vaccine.

### Inclusion criteria

Participants included women who were in their second or third trimester of pregnancy between December 2009 and March 2010, and had been offered the choice of receiving the monovalent A (H1N1) pdm09 vaccine during their pregnancy. At each study site, vaccinated and unvaccinated women participated in separate FGDs and IDIs.

### Data collection

Data was collected between October 4 and October 13, 2010. An experienced team with a background in conducting qualitative studies in Morocco was recruited including a facilitator, note-takers, and transcribers. The team was given a three-day initiation workshop on the purpose of the study, how to use the question guides and research ethics. Prior to the start of the study, the MOH study coordinator recruited participants by phone or door-to-door using a list of all patients receiving antenatal care at each facility. Participation was based on each woman's availability and interest to join either a FGD or IDI. The duration of each FGD was between 45–60 minutes, while each IDI took 20–30 minutes. All FGDs and IDIs were audio-recorded. The teams completed two FGD and two IDIs daily. For quality control, facilitators and study investigators met after each FGD to compare their notes and impressions. A summary of the day's events and any striking impressions from the facilitator were also documented on a daily basis to account for any bias. At the end of each working day, audio files were translated from Moroccan Arabic to English and transcribed. A bi-lingual member of the team made random quality checks on translations and transcriptions. The transcribed files were read daily by two study investigators to ensure that data collection remained focused on study objectives.

### Data analysis

As this was an applied qualitative study, thematic analysis was selected to optimize the provision of practical recommendations for program purposes [25]. After conducting a literature review, a list of the most common topics related to the public's acceptance of a new vaccine was established. An open-ended question guide with a set of probes was developed to ensure systematic coverage of the main topics that were identified in the literature review, namely (1) Knowledge, perceptions and risks related to the monovalent A (H1N1) pdm09 vaccine and A (H1N1) pandemic, (2) vaccine service related factors, (3) social factors, and (4) information sources.

First, two investigators independently read the transcripts several times to capture initial meanings and patterns as they related to factors that encourage and discourage vaccine uptake and the sources of information that influenced the decision making process of pregnant women. Second, data was coded independently by the same two investigators by highlighting words, phrases and sentences, which were then reviewed jointly to create the final set of codes by consensus. One investigator continued by cutting the highlighted sentences from the transcripts that summarized their responses to the codes and developed a chart onto which the relevant codes were sorted. The codes were merged into larger categories that lead to a set of themes. These themes were revised jointly by the two study investigators to ensure that they sufficiently answered the research questions. In the final stage of the analysis, the first author of this study identified the key characteristics and made the final interpretation of the data set as whole.

### Ethics statement

The facilitator obtained verbal consent from the participants before the start of each FGD and IDI, which was documented via audio recording. Verbal consent was considered appropriate due to the anticipated illiteracy of some participants and because verbal consent was the only approach linking participants with the study.

The study was reviewed and approved, including the verbal consent process, by the Ethical Review Board of the Ministry of Health in Morocco. The study was determined as non-research by the Institutional Review Boards at Centers for Disease Control and the Naval Medical Research Unit No. 3 in compliance with all applicable federal regulations governing the protection of human subjects.

## Results

### Study respondents

The sample included seven FGDs with 67 vaccinated women and another seven FGDs with 56 unvaccinated women. In addition, the sample included 8 IDIs distributed equally between vaccinated and unvaccinated women. Although no identifiable information was collected during the study, patients that utilize MOH facilities in these regions are typically of a low socioeconomic level.

### Perceptions of the A (H1N1) pdm09 pandemic

The analysis identified specific themes around the A (H1N1) pdm09 pandemic that related to severity of disease, signs and symptoms, modes of transmission, prevention, and etiology. “*Al Khanazir*” in Moroccan Arabic, directly translated as “swine flu,” was considered a severe and dangerous disease. Both vaccinated and unvaccinated participants shared a great fear of the unknown effects and lethal nature of the disease.

*“I was afraid I would lose my baby. I was scared that my baby would die **in** my belly **or** that I would pass away because of it* [A (H1N1) pdm09 virus].” (Vaccinated woman, Dar Bouzza)

Respondents also had unified understandings of the signs and symptoms of *influenza Al Khanazir*, which included a fever above 40 C, cough, sneezing and red eyes. Most respondents believed that *influenza Al Khanazir* was similar to seasonal influenza but more powerful and longer-lasting. The highly infectious nature of the disease was also well understood. Respondents commonly mentioned that preventive measures like hand-washing, utilization and proper disposal of tissues, not sharing utensils or cups, wearing masks and avoiding crowded places (bath houses, buses and schools) were techniques to avoid contracting *influenza Al Khanazir*.

It was widely believed that *influenza Al Khanazir* was of foreign origin and caused by animals such as pigs, chickens and donkeys. Respondents explained that *influenza A*l *Khanazir* came from Western countries or countries where people ate pigs such Europe, the US, Spain, Italy and Mexico. The women believed that foreign tourists, wealthy Moroccans that traveled abroad and soldiers brought *influenza Al Khanazir* into Morocco.

*“Europe is the source of all calamities, but for us we don't have any illness in Morocco thank God.”* (Vaccinated woman, Beb Fes)

### Fear of the influenza Al Khanazir

“We hear it is dangerous and we hear nobody knows what it can do.”

“They keep telling us in the television that it is dangerous and people died. Nobody knows what it can do us. God help us all.”

“It is worrying us because we did not hear about it before. Why did it appear now and how dangerous it will turn out. We used to have influenza but they say that this is something else.”

“Of course I am worried about the influenza Al Khanazir. But I don't know what it can do to me or to my unborn baby.”

“This influenza is worrying us all because they haven't done much research on it. So nobody knows much about it. We don't know how to protect and nobody can tell us what to do.”

“I don't know much about it but I know that it is really dangerous and it can lead to death.”

“They say it is dangerous. But what can it do. We don't know.”

“We heard that people died so it is a very dangerous infection.”

“I am afraid of the influenza of Al khanazir because we don't know what it does. But it can kill. We heard of death cases here in Morocco.”

“I think people were paralyzed and people got very sick. This infection has many dangerous consequences.”

“I know that this infection is new. So we don't know what can happen and what it can do. I hope my children will be safe.”

### Reasons influencing non-acceptance of the monovalent A (H1N1) pdm09 vaccine

Five main themes emerged surrounding non-acceptance of the vaccine: (1) Fear of the monovalent A (H1N1) pdm09 vaccine, (2) belief in an A (H1N1) pdm09 pandemic conspiracy, (3) belief in the inapplicability of the monovalent A (H1N1) pdm09 vaccine to Moroccans, (4) lack of knowledge of the monovalent A (H1N1) pdm09 vaccine, and (5) challenges of vaccination services/logistics.

Both unvaccinated and vaccinated women expressed similar fears surrounding the monovalent A (H1N1) pdm09 vaccine. Some simply feared that the vaccine might negatively impact their health and that of their infant. However, unvaccinated women often described their fears in more detail, explaining that the vaccine was to be feared because pigs were the origin of the *influenza Al Khanazir* virus and that the vaccine was associated with death and severe complications such as paralysis, cancer, and weakening of bones or body immunity systems. Moreover, respondents also believed that the vaccine may cause infertility or miscarriage. Respondents frequently explained the desire to have many more children and could not therefore risk taking the *influenza Al Khanazir* vaccine.

Respondents discussed their suspicions toward the vaccine in terms of conspiracy theories that were linked to financial and political interests. They suspected that *influenza Al Khanazir* did not exist because they had not seen anyone with the virus. Others believed that *influenza Al Khanazir* was created by the pharmaceutical companies to sell or test vaccines, or that the vaccine was imposed on Arabs because of the financial crisis that hit the US and Europe. The vaccine was also perceived as harmful for Muslims or even an attempt by Americans to harm Muslims.

*“… we talked about it but they said that this disease is nothing but a rumor…”* (Unvaccinated woman, Dar Bouzza)

Belief that the *influenza Al Khanazir* vaccine was not appropriate for Moroccans was also widespread. Participants thought that because the disease came from abroad from countries that had pigs, and since Moroccans did not eat pigs, there was no need to take the vaccine. Instead, it was important to keep a distance from foreigners and from those who had been abroad.

*“They say it is coming from foreigners, so why should we take it.”* (Unvaccinated woman, Beb Fes)
*“I see no benefit from this vaccine. Only foreigners can benefit from it that's why they sent it to us.”* (Unvaccinated woman, Beb Fes)

All FGDs with unvaccinated respondents included respondents who had not heard of the *influenza Al Khanazair* vaccine at all or had only heard the name of the vaccine but did not know any details. Some respondents believed that if they had known more about the vaccine, they would have considered vaccination.

*“I did not hear anything about this vaccine. I did not know people were taking it.”* (Unvaccinated respondent, Bernoussi)

At one interview site, several participants explained that they were willing to take the *influenza Al Khanazir* vaccine but they arrived too late on the vaccination day and missed the opportunity to get into the health center. In addition, one respondent claimed missing her vaccination appointment due to family circumstances the day of the vaccination.

### Factors influencing acceptance of the monovalent A (H1N1) pdm09 vaccine

Themes surrounding acceptance of the monovalent A (H1N1) pdm09 vaccine included perceived health benefits and modeling. Respondents were likely to accept the vaccine if they were convinced of its benefits. Vaccinated respondents believed that the vaccine gave them important protection and it was good for them and their baby. It was believed to protect them from *influenza Al Khanazir* and the seasonal flu and provide them with better health overall.

*“I wanted to be protected and I wanted to feel safe, so I went and took the vaccine.”* (Vaccinated woman, Hassania)

Respondents were encouraged get vaccinated when they heard that others had done so. Respondents explained having made the decision after hearing that everyone going to the pilgrimage (*hajj*) were vaccinated, and that soldiers, students and people abroad had taken the vaccine. In addition, respondents explained that their decision to vaccinate was based on the fact that everyone in their community had already been vaccinated or that they saw many others being vaccinated in the hospital.

*“… so I decided to take the vaccine at last, especially because many people took it here in Morocco like soldiers and also some people abroad.”* (Vaccinated woman, Ben Mansour)

### Decision making

The following information sources influenced the decisions made by pregnant women about *influenza Al Khanazir*: (1) family and husband, (2) neighbors, friends, community, (3) mass media, (4) religious leaders, and (5) health providers.

Family discussions often influenced the vaccine decision making process. If family members thought that the *influenza Al Khanazir* vaccine was beneficial, respondents were likely to vaccinate. However, family members often had divided opinions regarding the need to vaccinate and possible side effects, which confused respondents and made decision making more difficult. If the vaccine was taken without family consultation, respondents were likely to be blamed afterward for harming themselves and their unborn child. The husband was the most influential family member in the decision making process. Only one vaccinated respondent claimed that she took the vaccine against her husband's will.

*“My husband told me not to come back home if I take the vaccine.”* (Vaccinated woman, Ain Chock)

Discussions with neighbors and friends frequently fueled decisions not to vaccinate. These discussions were often based on rumors about complications and side effects affecting those who had been vaccinated. Respondents frequently mentioned the death of an individual in Casablanca which was repeated as a point of discussion among neighbors. Such discussions also created confusion among respondents.

*“We heard a lot of talk about it - in the street, from neighbors and family - nobody wanted the vaccine.”* (Unvaccinated woman, Kenitra)

Television was mentioned as a source of positive messages related to the *influenza Al Khanazir* vaccine. However, only one respondent claimed to have made the decision to vaccinate based solely on the information received through television.

*“We heard on TV that people must take this vaccine so I came here to take it.”* (Vaccinated woman, Bernoussi)

Only a few respondents had heard about the *influenza Al Khanazir* vaccine through Friday prayer or through announcements at local mosques. However, all those who received information via this route opted for vaccination.

*“I heard from the mosque and the next day I went to get the vaccine against H1N1.”* (Vaccinated woman, Ben Mansour)

Respondents explained that discussions with health providers were a powerful tool to convince them to vaccinate. Some respondents opted to take the *influenza Al Khanazir* vaccination without consulting their family if the health provider sufficiently explained the importance of the vaccine. Although some providers stressed the essential nature of the vaccine and others discussed the choice around vaccination, both types of provider interaction encouraged vaccine uptake. One vaccinated respondent explained that healthcare staff made her scared of serious complications such as paralysis if she did not vaccinate. Some unvaccinated respondents complained that health providers had not explained anything about the vaccine or had advised them not to vaccinate.

*“I heard that it had many risks and also that it had a bad effect on a pregnant women and her baby. When I came to the hospital, I was told that I should take the vaccine because it's good for pregnant women… so I took it.”* (Vaccinated woman, Kenitra)
*“We didn't know why we should take this vaccine. They (healthcare staff) just said take it, so we got afraid and we didn't take it as we didn't understand anything.”* (Unvaccinated woman, Sidi Yahya)
*“My doctor told me not to take the vaccine because I am pregnant and nobody knows the disadvantages of this vaccine.”* (Unvaccinated woman, Kinatra)

## Discussion

In the face of the A (H1N1) pdm09 pandemic, the Moroccan MOH initiated a health communication campaign that successfully informed pregnant women about the contagious nature of the infection, the potential lethality of the disease, and options for personal protection including vaccination. Despite these successful efforts and positive support from health providers communicating information and recommendations, vaccination rates remained much lower than expected. Rumors, conspiracy theories and misconceptions related to the origin of the A (H1N1) pdm09 pandemic negatively influenced vaccine uptake and, as such, must be addressed in future communication campaigns. Rumors often led study participants to believe that risks of the monovalent A (H1N1) pdm09 vaccine were high, that risk of infection was low, and that individuals had control over whether they were infected based on personal behavior. Recent studies in the US, Canada, Spain, France and Hong Kong indicate similar risk perceptions related to the monovalent A (H1N1) pdm09 vaccine [9], [11]–[23]. Rumors were a powerful negative influence on vaccine uptake and were reinforced by perceptions related to colonial imperialism and capitalism as the vaccine originated from the West. Similar findings are reported in a study conducted in Turkey where a considerable number of participants believed that the West was testing the efficacy of the vaccine in Turkey [26]. Rumors were also fueled by religious perceptions as the vaccine and virus were believed to be related, respectively, to pigs and eating pork, an act banned by Islam.

Future influenza communication campaigns could be improved by focusing on the creation of culturally acceptable ways to communicate the risk of infection and the importance of the vaccine. Implementation of methods to identify and rapidly respond to rumors would help to avoid more widespread misconceptions. Health authorities might also consider sending out regular updates on infection rates and vaccine safety to enable the public to reach rational conclusions about their individual level of risk of infection and the vaccine's risk/benefit ratio. New vaccines may be more likely to be accepted and conspiracy theories diminished if vaccine development is seen as a combined effort of the Ministries of Health in various countries working with the international health community. Communication should also help the public better understand that influenza infection occurs regardless of geographic location, financial situation, or cultural and religious identity.

In addition, rumors were also linked to specific fears related to pregnancy and child bearing; both of high importance in Moroccan society where motherhood continues to define the role and status of women, and children are at the center of life. Unique communication needs surrounding the A (H1N1) pdm09 pandemic in pregnant women have been previously established in studies among pregnant women in the US and in Canada [11], [12], [17], [27], [28], [29], [30]. Messages directing pregnant women to adopt influenza vaccine recommendations should include detailed pregnancy-specific descriptions of the risk/benefit ratio for the fetus. Messages should also address concerns related to possible long-term side effects of the influenza related to pregnant women. Additionally, messages should recognize that pregnant women are taught to be selective about taking medication and provide a clear rationale as to why they should choose to vaccinate.

Study participants were strongly influenced by health providers, their families, neighbors, and religious leaders when making decisions regarding uptake of the monovalent A (H1N1) pdm09 vaccine. Study respondents trusted the opinions and advice of health providers and claimed to have made their vaccination choice based on their recommendations. In general, where vaccination was successful, health staff did not coerce compliance but rather helped their patients make an educated choice. Recent studies elsewhere also conclude that health providers play a key role in the success of vaccine campaigns [29], [31] and in conveying vaccine-related messages [32], [33]. Provider knowledge of the monovalent A (H1N1) pdm09 vaccine has been shown to correlate with improved vaccine uptake in other countries [14], [17], [21], [34], which further highlights the importance educating health providers about influenza and vaccination. Although the MoH in Morocco provided informative workshops for health officials on a regional level during the A (H1N1) pdm09 pandemic, additional provider level training would likely have a positive impact on vaccine uptake.

The decision-making process of study respondents was mainly active and was based on advice-seeking behavior including from family and community at large, suggesting that broad communication efforts that target other sub-groups as advocates for vaccine uptake may also be effective. To maximize effectiveness, vaccine communication campaigns may need to include influential figures such as religious leaders and teachers. Public health officials should also anticipate the need for active decision making for pregnant women as this process requires sufficient time prior to initiation of the scheduled vaccination campaign.

Our study methodology was based on a mix of two different tools to obtain information, which ensured that readily discussed topics in a group setting and more sensitive topics in individual interviews were captured. Although the study sample is somewhat limited, it does represent both rural and urban populations in geographically distinct regions of Morocco. However, this study does have limitations. Selection bias may have occurred during recruitment as eligible mothers with negative perceptions of the vaccine may have been less likely to agree to participate. Participants' reasons for accepting or refusing the monovalent A (H1N1) pdm09 vaccine might have been subject to biases related to social desirability as the IDIs and FGDs were conducted in health centers where women receive their pregnancy-related health services. Factors related to service provision and other less socially acceptable reasons may have been under reported. This bias stems from a propensity to report “real” motivation is well documented in the methodological literature [35]. Despite these possible pitfalls, our findings are consistent with existing international literature discussing uptake of the monovalent A (H1N1) pdm09 vaccine [9], [12]–[15].

Lessons learned from this qualitative study can inform a well-grounded communication strategy for future vaccine campaigns targeting pregnant women in Morocco. Vaccination acceptance was positively influenced when women clearly understood the benefits of the monovalent A (H1N1) pdm09 vaccine and when vaccination was perceived as a social norm. Cultural beliefs, values and preference play an important role in the decision making process and must be considered when communicating with pregnant women. Communication efforts must address rumors and pregnancy-specific concerns. In addition, family, health providers and religious leaders who influence the decision making of pregnant women must be included in campaign plans. As cultural and religious values are shared across many Arab countries, these findings may also provide valuable insights for seasonal influenza vaccine planning in the Middle East and North Africa region at large. Finally, this study highlights the need for culturally specific qualitative research on norms, perceptions and beliefs as well as cultural factors linked with behavior change prior to the introduction of any novel vaccine in order to optimize public health communication strategies.
